# Bis(1*H*-imidazole-κ*N* ^3^){2,2′-[propane-1,2-diylbis(nitrilo­methyl­idyne)]diphenolato-κ^4^ *O*,*N*,*N*′,*O*′}iron(III) perchlorate

**DOI:** 10.1107/S1600536810004010

**Published:** 2010-02-17

**Authors:** Yoshihiro Kojima, Kazuya Kato, Yuuki Yamamoto, Katsuya Inoue, Shinya Hayami

**Affiliations:** a Department of Chemistry, Graduate School of Science, Hiroshima University, 1-3-1 Kagamiyama, Higashi-Hiroshima, 739-8526, Japan; b Department of Chemistry, Graduate School of Science and Technology, Kumamoto University, 2-39-1 Kurokami, Kumamoto, 860-8555, Japan

## Abstract

The title compound, [Fe(C_17_H_16_N_2_O_2_)(C_3_H_4_N_2_)_2_]ClO_4_, consists of monomeric [Fe(salmen)(HIm)_2_]^+^ cations {salmen is the 2,2′-[propane-1,2-diylbis(nitrilo­methyl­idyne)]diphen­olate dianion and HIm is 1*H*-imiazole} and perchlorate anions. In the cation, the Fe^3+^ ion is octahedrally coordinated by two N atoms and two O atoms from a tetra­dentate salmen anion and two N atoms from two Him mol­ecules. These ligands are coordinated to the iron ion in a direction perpendicular to the [Fe(salmen)]^+^ coordination plane. The benzene ring planes in the salmen ligands are oriented nearly parallel to one another inter­molecularly [dihedral angle = 6.36 (3)°]. The dihedral angle between the mean planes through the imidazole rings in the cation is 76.9 (2)°. In the crystal, N—H⋯O inter­actions link the mol­ecules into a one-dimensional double chain running along [101] and C—H⋯O inter­actions link the double chains into a two-dimensional network, running parallel to the *ac* plane.

## Related literature

For salen–metal complexes with spin crossover properties, see: Brendan *et al.* (1984 ▶, 1987 ▶); Hernández-Molina *et al.* (1998 ▶).
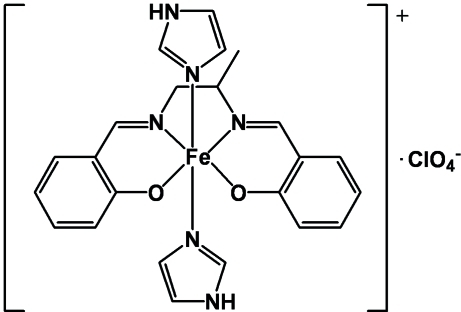


## Experimental

### 

#### Crystal data

[Fe(C_17_H_16_N_2_O_2_)(C_3_H_4_N_2_)_2_]ClO_4_
*M* *_r_* = 571.78
Monoclinic, 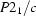

*a* = 10.4898 (8) Å
*b* = 16.4312 (9) Å
*c* = 14.7729 (8) Å
β = 105.5081 (17)°
*V* = 2453.6 (3) Å^3^
*Z* = 4
Mo *K*α radiation
μ = 0.78 mm^−1^
*T* = 113 K
0.20 × 0.20 × 0.20 mm

#### Data collection

Rigaku R-AXIS RAPID Imaging Plate diffractometer
Absorption correction: multi-scan (*ABSCOR*; Higashi, 2001 ▶) *T*_min_ = 0.860, *T*_max_ = 0.860
20794 measured reflections
5615 independent reflections
3529 reflections with *I* > 2σ(*I*)
*R*_int_ = 0.086

#### Refinement

*R*[*F* ^2^ > 2σ(*F* ^2^)] = 0.065
*wR*(*F* ^2^) = 0.179
*S* = 0.99
5615 reflections
335 parameters
H-atom parameters constrained
Δρ_max_ = 0.77 e Å^−3^
Δρ_min_ = −0.65 e Å^−3^

### 

Data collection: *PROCESS-AUTO* (Rigaku, 1998 ▶); cell refinement: *PROCESS-AUTO*; data reduction: *CrystalClear* (Molecular Structure Corporation and Rigaku, 2002 ▶); program(s) used to solve structure: *SHELXS97* (Sheldrick, 2008 ▶); program(s) used to refine structure: *SHELXL97* (Sheldrick, 2008 ▶); molecular graphics: *Yadokari-XG* (Wakita, 2000 ▶); software used to prepare material for publication: *SHELXL97*.

## Supplementary Material

Crystal structure: contains datablocks I, global. DOI: 10.1107/S1600536810004010/kj2139sup1.cif

Structure factors: contains datablocks I. DOI: 10.1107/S1600536810004010/kj2139Isup2.hkl

Additional supplementary materials:  crystallographic information; 3D view; checkCIF report

## Figures and Tables

**Table 1:** Selected bond lengths (Å)

Fe1—O1	1.879 (2)
Fe1—O2	1.914 (3)
Fe1—N1	2.119 (3)
Fe1—N2	2.108 (3)
Fe1—N3	2.161 (3)
Fe1—N5	2.161 (3)

**Table 2:** Hydrogen-bond geometry (Å, °)

*D*—H⋯*A*	*D*—H	H⋯*A*	*D*⋯*A*	*D*—H⋯*A*
C7—H7⋯O6^i^	0.95	2.53	3.436 (6)	161
C16—H16⋯O5	0.95	2.53	3.325 (6)	142
N4—H4*A*⋯O2^ii^	0.88	2.48	3.063 (4)	125
N4—H4*A*⋯O6^ii^	0.88	2.36	3.031 (4)	133
N6—H6*A*⋯O4^iii^	0.88	2.03	2.892 (4)	167

Symmetry codes: (i) 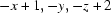; (ii) 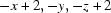; (iii) 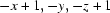.

**Fig. 1.**
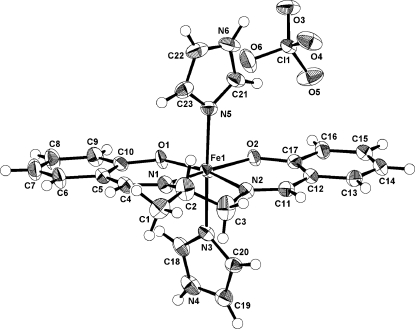
The molecular structure of the title compound drawn with 50% probability displacement ellipsoids.

## supplementary crystallographic information

### Comment

A number of spin-crossover compounds have been studied. The salen molecule (salen = *N,N'*-ethylenebis(salicylideneiminato) dianion) has often been used as ligand in spin-crossover complexes (Brendan *et al.*, 1984, Hernández-Molina *et al.*, 1998). Brendan *et al.* reported Fe(III)-salen complexes [Fe(salen)(L)_2_](Y) (L = imidazole series, Y = counter anion) and showed that the spin state can be tuned by using different imidazole series and counter anions (Brendan *et al.*, 1987). They also showed that [Fe(salen)(HIm)_2_](ClO_4_)_2_ has spin-crossover properties. In this study, the crystal stucture of the derivative [Fe(salmen)(HIm)_2_](ClO_4_)_2_ is reported.

The title compound consists of a cation whose iron ion is coordinated by a salmen anion and two imidazole ligands. The structure further contains a perchlorate anion. The molecular planes of the benzene rings of all salmen ligands in the crystal are oriented essentially parallel to one another. The two imidazoles coordinated to the Fe^3+^ ion aren't coplanar; the dihedral angle between their mean planes is 76.9 (2)°. Imidazole ligands are coordinated to iron ion in a direction perpendicular to [Fe(salmen)]^+^, with the angle around iron ion O1— Fe1— N3 = 88.40 (12)°, O2—Fe1—N3 = 90.95 (12)°, O1—Fe1—N5 = 93.19 (12)° and O2—Fe1—N5 = 87.99 (12)°. The two benzene rings in a salmen ligand are nearly coplanar, but the bridging carbon atoms are not located in this plane. C2 is displaced 0.1057 (2)Å from the C5–C12 benzene plane and C3 is displaced 0.1785 (2)Å from the C12–C17 benzene plane. The torsion angle N1—C2—C3—N2 is 41.1 (5)°.

In addition, many intermolecular interactions are observed in the crystal structure. Intermolecular C—H···O hydrogen bonds link the benzene hydrogens H7 and H16 with the anion oxygens O6 and O5, respectively. N—H···O hydrogen bonds link the imidazole hydrogen H6A to anionic oxygen O4 and link the imidazole H4A in a bifurcated bond to the ring oxygen O2 and the anion oxygen O6. The N—H···O interactions link the molecules into a one-dimensional double chain (step ladder) running in the [1 0 1] direction, with N4—H4A···O2 acting as the rungs in the ladder. The C—H···O interactions link the double chains into a two-dimensional network, running parallel to the *ac* plane.

### Experimental

The salmen ligand was prepared by the reaction of 1, 2-diaminopropane (2 mmol) and salicylaldehyde (4 mmol) in ethanol. The title compound was synthesized in accordance with the procedure reported in the literature (Brendan *et al*., 1987).

### Refinement

All H-atoms were positioned geometrically (N—H = 0.88 Å and C—H = 0.95 – 0.99Å ) and refined a riding model with *U*_iso_(H) = 1.2 *U*_eq_(C, N) or 1.5 *U*_eq_(C) for methyl H atoms.

### Crystal data

**Table:**

[Fe(C_17_H_16_N_2_O_2_)(C_3_H_4_N_2_)_2_]ClO_4_	*F*(000) = 1180
*M**_r_* = 571.78	*D*_x_ = 1.548 Mg m^−^^3^
Monoclinic, *P*2_1_/*c*	Mo *K*α radiation, λ = 0.71069 Å
*a* = 10.4898 (8) Å	Cell parameters from 18352 reflections
*b* = 16.4312 (9) Å	θ = 2.5–27.5°
*c* = 14.7729 (8) Å	µ = 0.78 mm^−^^1^
β = 105.5081 (17)°	*T* = 113 K
*V* = 2453.6 (3) Å^3^	Block, black
*Z* = 4	0.20 × 0.20 × 0.20 mm

### Data collection

**Table:**

Rigaku R-AXIS RAPID Imaging Plate diffractometer	5615 independent reflections
Radiation source: fine-focus sealed tube	3529 reflections with *I* > 2σ(*I*)
graphite	*R*_int_ = 0.086
ω scans	θ_max_ = 27.5°, θ_min_ = 2.4°
Absorption correction: multi-scan (*ABSCOR*; Higashi, 2001)	*h* = −13→11
*T*_min_ = 0.860, *T*_max_ = 0.860	*k* = −21→21
20794 measured reflections	*l* = −19→19

### Refinement

**Table:**

Refinement on *F*^2^	Primary atom site location: structure-invariant direct methods
Least-squares matrix: full	Secondary atom site location: difference Fourier map
*R*[*F*^2^ > 2σ(*F*^2^)] = 0.065	Hydrogen site location: inferred from neighbouring sites
*wR*(*F*^2^) = 0.179	H-atom parameters constrained
*S* = 0.99	*w* = 1/[σ^2^(*F*_o_^2^) + (0.1*P*)^2^] where *P* = (*F*_o_^2^ + 2*F*_c_^2^)/3
5615 reflections	(Δ/σ)_max_ = 0.001
335 parameters	Δρ_max_ = 0.77 e Å^−^^3^
0 restraints	Δρ_min_ = −0.65 e Å^−^^3^

### Special details

**Table:**

Geometry. All esds (except the esd in the dihedral angle between two l.s. planes) are estimated using the full covariance matrix. The cell esds are taken into account individually in the estimation of esds in distances, angles and torsion angles; correlations between esds in cell parameters are only used when they are defined by crystal symmetry. An approximate (isotropic) treatment of cell esds is used for estimating esds involving l.s. planes.
Refinement. Refinement of *F*^2^ against ALL reflections. The weighted *R*-factor wR and goodness of fit *S* are based on *F*^2^, conventional *R*-factors *R* are based on *F*, with *F* set to zero for negative *F*^2^. The threshold expression of *F*^2^ > σ(*F*^2^) is used only for calculating *R*-factors(gt) etc. and is not relevant to the choice of reflections for refinement. *R*-factors based on *F*^2^ are statistically about twice as large as those based on *F*, and *R*- factors based on ALL data will be even larger.

### Fractional atomic coordinates and isotropic or equivalent isotropic displacement parameters (Å^2^)

**Table:**

	*x*	*y*	*z*	*U*_iso_*/*U*_eq_	
Fe1	0.79757 (6)	0.13608 (3)	0.88002 (3)	0.02225 (18)	
Cl1	0.69936 (10)	−0.13827 (6)	0.61401 (6)	0.0306 (2)	
O1	0.7346 (3)	0.06289 (15)	0.95574 (17)	0.0267 (6)	
O2	0.8686 (3)	0.06897 (16)	0.79939 (17)	0.0268 (6)	
O3	0.6186 (3)	−0.20419 (19)	0.5683 (2)	0.0464 (9)	
O4	0.6768 (4)	−0.06891 (19)	0.5516 (2)	0.0506 (9)	
O5	0.8363 (3)	−0.1597 (2)	0.6359 (3)	0.0530 (10)	
O6	0.6683 (4)	−0.1151 (3)	0.6984 (2)	0.0633 (11)	
N1	0.7220 (3)	0.23386 (19)	0.9436 (2)	0.0283 (8)	
N2	0.8607 (3)	0.23941 (19)	0.8192 (2)	0.0258 (7)	
N3	0.9857 (3)	0.13642 (19)	0.9853 (2)	0.0266 (7)	
N4	1.1404 (4)	0.0971 (2)	1.1086 (2)	0.0386 (9)	
H4A	1.1795	0.0723	1.1615	0.046*	
N5	0.6125 (3)	0.1369 (2)	0.7717 (2)	0.0276 (8)	
N6	0.4654 (4)	0.1116 (2)	0.6386 (2)	0.0400 (10)	
H6A	0.4292	0.0916	0.5823	0.048*	
C1	0.7007 (5)	0.3882 (3)	0.9504 (3)	0.0436 (12)	
H1A	0.6109	0.3875	0.9584	0.065*	
H1B	0.7123	0.4370	0.9153	0.065*	
H1C	0.7651	0.3887	1.0122	0.065*	
C2	0.7217 (5)	0.3135 (3)	0.8970 (3)	0.0437 (12)	
H2	0.6444	0.3116	0.8401	0.052*	
C3	0.8397 (5)	0.3177 (3)	0.8607 (4)	0.0465 (13)	
H3A	0.8276	0.3610	0.8125	0.056*	
H3B	0.9183	0.3314	0.9125	0.056*	
C4	0.6667 (4)	0.2260 (2)	1.0119 (3)	0.0292 (9)	
H4	0.6375	0.2744	1.0354	0.035*	
C5	0.6456 (4)	0.1508 (3)	1.0550 (3)	0.0289 (9)	
C6	0.5831 (5)	0.1545 (3)	1.1286 (3)	0.0368 (11)	
H6	0.5582	0.2062	1.1472	0.044*	
C7	0.5574 (5)	0.0875 (3)	1.1733 (3)	0.0429 (12)	
H7	0.5132	0.0920	1.2214	0.051*	
C8	0.5962 (5)	0.0116 (3)	1.1484 (3)	0.0420 (12)	
H8	0.5811	−0.0357	1.1811	0.050*	
C9	0.6569 (5)	0.0047 (3)	1.0759 (3)	0.0347 (10)	
H9	0.6829	−0.0474	1.0595	0.042*	
C10	0.6801 (4)	0.0724 (2)	1.0274 (2)	0.0270 (9)	
C11	0.9182 (4)	0.2386 (3)	0.7521 (3)	0.0283 (9)	
H11	0.9369	0.2898	0.7285	0.034*	
C12	0.9565 (4)	0.1668 (3)	0.7100 (3)	0.0278 (9)	
C13	1.0235 (4)	0.1789 (3)	0.6396 (3)	0.0335 (10)	
H13	1.0400	0.2329	0.6226	0.040*	
C14	1.0649 (5)	0.1152 (3)	0.5955 (3)	0.0375 (11)	
H14	1.1102	0.1247	0.5487	0.045*	
C15	1.0401 (4)	0.0360 (3)	0.6199 (3)	0.0363 (11)	
H15	1.0677	−0.0087	0.5890	0.044*	
C16	0.9763 (4)	0.0220 (3)	0.6880 (3)	0.0340 (10)	
H16	0.9611	−0.0325	0.7039	0.041*	
C17	0.9327 (4)	0.0864 (3)	0.7351 (3)	0.0277 (9)	
C18	1.0139 (5)	0.0901 (3)	1.0620 (3)	0.0320 (10)	
H18	0.9518	0.0566	1.0808	0.038*	
C19	1.1997 (5)	0.1494 (3)	1.0607 (3)	0.0355 (10)	
H19	1.2899	0.1658	1.0774	0.043*	
C20	1.1030 (4)	0.1732 (2)	0.9839 (3)	0.0286 (9)	
H20	1.1147	0.2095	0.9368	0.034*	
C21	0.5883 (4)	0.0979 (3)	0.6904 (3)	0.0327 (10)	
H21	0.6506	0.0645	0.6715	0.039*	
C22	0.4052 (5)	0.1619 (3)	0.6876 (3)	0.0414 (12)	
H22	0.3171	0.1819	0.6678	0.050*	
C23	0.4955 (4)	0.1780 (3)	0.7700 (3)	0.0336 (10)	
H23	0.4814	0.2117	0.8186	0.040*	

### Atomic displacement parameters (Å^2^)

**Table:**

	*U*^11^	*U*^22^	*U*^33^	*U*^12^	*U*^13^	*U*^23^
Fe1	0.0297 (3)	0.0202 (3)	0.0168 (3)	−0.0031 (3)	0.0062 (2)	0.0003 (2)
Cl1	0.0300 (6)	0.0369 (6)	0.0223 (5)	−0.0017 (5)	0.0027 (4)	−0.0049 (4)
O1	0.0457 (18)	0.0202 (14)	0.0196 (13)	−0.0033 (12)	0.0179 (12)	−0.0002 (11)
O2	0.0360 (17)	0.0266 (15)	0.0207 (13)	−0.0046 (12)	0.0125 (12)	−0.0028 (11)
O3	0.046 (2)	0.0410 (19)	0.0427 (18)	−0.0176 (16)	−0.0051 (15)	0.0030 (15)
O4	0.068 (3)	0.0364 (19)	0.0353 (17)	−0.0089 (17)	−0.0065 (16)	0.0077 (15)
O5	0.032 (2)	0.0399 (19)	0.081 (3)	0.0024 (16)	0.0046 (18)	−0.0156 (18)
O6	0.062 (3)	0.102 (3)	0.0309 (17)	0.008 (2)	0.0201 (17)	−0.0083 (19)
N1	0.038 (2)	0.0223 (18)	0.0258 (17)	−0.0004 (15)	0.0100 (15)	0.0028 (14)
N2	0.033 (2)	0.0216 (17)	0.0227 (16)	−0.0018 (14)	0.0071 (15)	0.0003 (14)
N3	0.034 (2)	0.0221 (17)	0.0214 (16)	0.0028 (15)	0.0039 (14)	−0.0011 (14)
N4	0.044 (2)	0.041 (2)	0.0259 (18)	0.0210 (19)	0.0008 (17)	0.0019 (17)
N5	0.029 (2)	0.0269 (18)	0.0247 (16)	−0.0055 (16)	0.0032 (14)	0.0011 (15)
N6	0.045 (3)	0.040 (2)	0.0264 (18)	−0.0146 (19)	−0.0063 (17)	0.0028 (17)
C1	0.042 (3)	0.036 (3)	0.054 (3)	0.001 (2)	0.015 (2)	0.001 (2)
C2	0.066 (4)	0.023 (2)	0.048 (3)	0.000 (2)	0.026 (3)	−0.001 (2)
C3	0.067 (4)	0.025 (2)	0.057 (3)	0.001 (2)	0.032 (3)	0.006 (2)
C4	0.034 (3)	0.029 (2)	0.024 (2)	0.0023 (18)	0.0075 (18)	−0.0048 (18)
C5	0.032 (2)	0.032 (2)	0.0224 (19)	−0.0017 (18)	0.0078 (17)	−0.0033 (17)
C6	0.050 (3)	0.039 (3)	0.029 (2)	0.002 (2)	0.021 (2)	−0.0055 (19)
C7	0.054 (3)	0.047 (3)	0.037 (2)	−0.007 (2)	0.028 (2)	−0.008 (2)
C8	0.063 (3)	0.037 (3)	0.033 (2)	−0.013 (2)	0.024 (2)	−0.001 (2)
C9	0.050 (3)	0.025 (2)	0.033 (2)	−0.007 (2)	0.017 (2)	−0.0014 (18)
C10	0.032 (2)	0.029 (2)	0.0188 (18)	−0.0061 (18)	0.0054 (17)	−0.0024 (17)
C11	0.032 (2)	0.028 (2)	0.0238 (19)	−0.0055 (18)	0.0044 (17)	0.0065 (17)
C12	0.027 (2)	0.034 (2)	0.0217 (19)	−0.0025 (18)	0.0056 (17)	−0.0007 (17)
C13	0.033 (3)	0.041 (3)	0.027 (2)	−0.008 (2)	0.0077 (19)	0.0062 (19)
C14	0.036 (3)	0.057 (3)	0.020 (2)	−0.004 (2)	0.0084 (19)	−0.002 (2)
C15	0.031 (3)	0.052 (3)	0.025 (2)	−0.003 (2)	0.0057 (19)	−0.006 (2)
C16	0.040 (3)	0.038 (3)	0.027 (2)	−0.006 (2)	0.0136 (19)	−0.0025 (19)
C17	0.027 (2)	0.035 (2)	0.0208 (19)	−0.0059 (18)	0.0045 (17)	0.0019 (17)
C18	0.045 (3)	0.028 (2)	0.023 (2)	0.010 (2)	0.0078 (19)	0.0044 (17)
C19	0.032 (3)	0.039 (3)	0.034 (2)	0.008 (2)	0.008 (2)	−0.005 (2)
C20	0.030 (2)	0.025 (2)	0.028 (2)	0.0017 (18)	0.0046 (18)	−0.0047 (17)
C21	0.039 (3)	0.031 (2)	0.024 (2)	−0.007 (2)	0.0010 (19)	0.0026 (18)
C22	0.030 (3)	0.041 (3)	0.045 (3)	−0.011 (2)	−0.003 (2)	0.014 (2)
C23	0.031 (3)	0.028 (2)	0.041 (2)	−0.0066 (19)	0.009 (2)	0.0048 (19)

### Geometric parameters (Å, °)

**Table:**

Fe1—O1	1.879 (2)	C3—H3B	0.9900
Fe1—O2	1.914 (3)	C4—C5	1.434 (6)
Fe1—N1	2.119 (3)	C4—H4	0.9500
Fe1—N2	2.108 (3)	C5—C6	1.412 (5)
Fe1—N3	2.161 (3)	C5—C10	1.428 (6)
Fe1—N5	2.161 (3)	C6—C7	1.348 (6)
Cl1—O3	1.428 (3)	C6—H6	0.9500
Cl1—O4	1.445 (3)	C7—C8	1.392 (6)
Cl1—O5	1.429 (3)	C7—H7	0.9500
Cl1—O6	1.422 (3)	C8—C9	1.389 (6)
O1—C10	1.339 (4)	C8—H8	0.9500
O2—C17	1.333 (4)	C9—C10	1.380 (6)
N1—C2	1.478 (5)	C9—H9	0.9500
N1—C4	1.297 (5)	C11—C12	1.441 (6)
N2—C3	1.466 (5)	C11—H11	0.9500
N2—C11	1.291 (5)	C12—C13	1.416 (5)
N3—C18	1.331 (5)	C12—C17	1.413 (6)
N3—C20	1.376 (5)	C13—C14	1.364 (6)
N4—C18	1.327 (5)	C13—H13	0.9500
N4—C19	1.364 (6)	C14—C15	1.394 (6)
N4—H4A	0.8800	C14—H14	0.9500
N5—C21	1.325 (5)	C15—C16	1.368 (6)
N5—C23	1.395 (5)	C15—H15	0.9500
N6—C21	1.331 (5)	C16—C17	1.408 (6)
N6—C22	1.360 (6)	C16—H16	0.9500
N6—H6A	0.8800	C18—H18	0.9500
C1—C2	1.507 (6)	C19—C20	1.361 (6)
C1—H1A	0.9800	C19—H19	0.9500
C1—H1B	0.9800	C20—H20	0.9500
C1—H1C	0.9800	C21—H21	0.9500
C2—C3	1.476 (7)	C22—C23	1.353 (6)
C2—H2	1.0000	C22—H22	0.9500
C3—H3A	0.9900	C23—H23	0.9500
			
O1—Fe1—O2	105.02 (11)	N1—C4—H4	117.1
O1—Fe1—N1	89.34 (12)	C5—C4—H4	117.1
O1—Fe1—N2	165.83 (12)	C4—C5—C6	117.6 (4)
O1—Fe1—N3	88.40 (12)	C4—C5—C10	124.7 (3)
O1—Fe1—N5	93.19 (12)	C6—C5—C10	117.7 (4)
O2—Fe1—N1	165.15 (12)	C5—C6—C7	122.4 (4)
O2—Fe1—N2	88.86 (12)	C5—C6—H6	118.8
O2—Fe1—N3	90.95 (12)	C7—C6—H6	118.8
O2—Fe1—N5	87.99 (12)	C6—C7—C8	119.5 (4)
N1—Fe1—N2	77.04 (12)	C6—C7—H7	120.3
N1—Fe1—N3	93.25 (13)	C8—C7—H7	120.3
N1—Fe1—N5	87.44 (13)	C7—C8—C9	120.2 (4)
N2—Fe1—N3	88.51 (12)	C7—C8—H8	119.9
N2—Fe1—N5	90.10 (12)	C9—C8—H8	119.9
N3—Fe1—N5	178.27 (12)	C8—C9—C10	121.1 (4)
O3—Cl1—O4	108.75 (19)	C8—C9—H9	119.5
O3—Cl1—O5	110.6 (2)	C10—C9—H9	119.5
O3—Cl1—O6	111.8 (2)	O1—C10—C9	119.3 (4)
O4—Cl1—O5	108.8 (2)	O1—C10—C5	121.7 (3)
O4—Cl1—O6	108.2 (2)	C5—C10—C9	119.0 (4)
O5—Cl1—O6	108.6 (2)	N2—C11—C12	125.6 (4)
Fe1—O1—C10	133.5 (2)	N2—C11—H11	117.2
Fe1—O2—C17	132.4 (3)	C12—C11—H11	117.2
Fe1—N1—C2	114.7 (2)	C11—C12—C13	116.9 (4)
Fe1—N1—C4	124.6 (3)	C11—C12—C17	124.3 (3)
C2—N1—C4	120.3 (3)	C13—C12—C17	118.8 (4)
Fe1—N2—C3	115.4 (3)	C12—C13—C14	121.8 (4)
Fe1—N2—C11	125.7 (3)	C12—C13—H13	119.1
C3—N2—C11	118.9 (3)	C14—C13—H13	119.1
Fe1—N3—C18	124.3 (3)	C13—C14—C15	119.2 (4)
Fe1—N3—C20	129.7 (3)	C13—C14—H14	120.4
C18—N3—C20	105.5 (4)	C15—C14—H14	120.4
C18—N4—C19	108.5 (4)	C14—C15—C16	120.6 (4)
C18—N4—H4A	125.8	C14—C15—H15	119.7
C19—N4—H4A	125.8	C16—C15—H15	119.7
Fe1—N5—C21	125.8 (3)	C15—C16—C17	121.6 (4)
Fe1—N5—C23	128.9 (3)	C15—C16—H16	119.2
C21—N5—C23	105.3 (4)	C17—C16—H16	119.2
C21—N6—C22	108.3 (4)	O2—C17—C12	123.1 (4)
C21—N6—H6A	125.9	O2—C17—C16	118.9 (4)
C22—N6—H6A	125.9	C12—C17—C16	118.0 (3)
C2—C1—H1A	109.5	N3—C18—N4	110.9 (4)
C2—C1—H1B	109.5	N3—C18—H18	124.6
C2—C1—H1C	109.5	N4—C18—H18	124.6
H1A—C1—H1B	109.5	N4—C19—C20	105.7 (4)
H1A—C1—H1C	109.5	N4—C19—H19	127.1
H1B—C1—H1C	109.5	C20—C19—H19	127.1
N1—C2—C1	117.3 (4)	N3—C20—C19	109.4 (4)
N1—C2—C3	108.2 (4)	N3—C20—H20	125.3
N1—C2—H2	105.4	C19—C20—H20	125.3
C1—C2—C3	114.0 (4)	N5—C21—N6	111.1 (4)
C1—C2—H2	105.4	N5—C21—H21	124.4
C3—C2—H2	105.4	N6—C21—H21	124.4
N2—C3—C2	110.2 (4)	N6—C22—C23	106.5 (4)
N2—C3—H3A	109.6	N6—C22—H22	126.7
N2—C3—H3B	109.6	C23—C22—H22	126.7
C2—C3—H3A	109.6	N5—C23—C23	108.8 (4)
C2—C3—H3B	109.6	N5—C23—H23	125.6
H3A—C3—H3B	108.1	C22—C23—H23	125.6
N1—C4—C5	125.8 (4)		
			
O2—Fe1—O1—C10	176.7 (3)	Fe1—N1—C2—C3	−35.1 (5)
N1—Fe1—O1—C10	−7.2 (4)	C4—N1—C2—C1	21.0 (6)
N2—Fe1—O1—C10	8.6 (8)	C4—N1—C2—C3	151.7 (4)
N3—Fe1—O1—C10	86.1 (4)	Fe1—N1—C4—C5	−0.8 (6)
N5—Fe1—O1—C10	−94.6 (4)	C2—N1—C4—C5	171.8 (4)
O1—Fe1—O2—C17	−175.8 (3)	Fe1—N2—C3—C2	−30.6 (5)
N1—Fe1—O2—C17	19.3 (7)	C11—N2—C3—C2	151.3 (4)
N2—Fe1—O2—C17	1.3 (3)	Fe1—N2—C11—C12	−3.7 (6)
N3—Fe1—O2—C17	−87.2 (3)	C3—N2—C11—C12	174.2 (4)
N5—Fe1—O2—C17	91.4 (3)	Fe1—N3—C18—N4	173.6 (3)
O1—Fe1—N1—C2	−169.1 (3)	C20—N3—C18—N4	1.0 (4)
O1—Fe1—N1—C4	3.9 (3)	Fe1—N3—C20—C19	−173.0 (3)
O2—Fe1—N1—C2	−3.6 (7)	C18—N3—C20—C19	−0.9 (4)
O2—Fe1—N1—C4	169.3 (4)	Fe1—N5—C23—C22	179.1 (3)
N2—Fe1—N1—C2	14.9 (3)	C19—N4—C18—N3	−0.8 (5)
N2—Fe1—N1—C4	−172.2 (4)	C18—N4—C19—C20	0.2 (5)
N3—Fe1—N1—C2	102.6 (3)	C21—N5—C23—C22	0.1 (5)
N3—Fe1—N1—C4	−84.5 (3)	Fe1—N5—C21—N6	−179.1 (3)
N5—Fe1—N1—C4	97.1 (3)	C23—N5—C21—N6	0.0 (4)
N5—Fe1—N1—C2	−75.8 (3)	C22—N6—C21—N5	0.0 (5)
O1—Fe1—N2—C3	−7.4 (7)	C21—N6—C22—C23	0.1 (5)
O1—Fe1—N2—C11	170.5 (4)	N1—C2—C3—N2	41.1 (5)
O2—Fe1—N2—C3	−175.8 (3)	C1—C2—C3—N2	173.6 (4)
O2—Fe1—N2—C11	2.1 (3)	N1—C4—C5—C6	−179.8 (4)
N1—Fe1—N2—C3	8.8 (3)	N1—C4—C5—C10	−1.6 (7)
N1—Fe1—N2—C11	−173.2 (4)	C4—C5—C6—C7	179.4 (4)
N3—Fe1—N2—C3	−84.9 (3)	C10—C5—C6—C7	1.0 (7)
N3—Fe1—N2—C11	93.1 (3)	C4—C5—C10—O1	−1.1 (6)
N5—Fe1—N2—C3	96.2 (3)	C4—C5—C10—C9	178.8 (4)
N5—Fe1—N2—C11	−85.9 (3)	C6—C5—C10—O1	177.1 (4)
O1—Fe1—N3—C18	4.1 (3)	C6—C5—C10—C9	−3.0 (6)
O1—Fe1—N3—C20	174.8 (3)	C5—C6—C7—C8	1.6 (8)
O2—Fe1—N3—C18	−100.9 (3)	C6—C7—C8—C9	−2.1 (8)
O2—Fe1—N3—C20	69.8 (3)	C7—C8—C9—C10	0.0 (7)
N1—Fe1—N3—C18	93.3 (3)	C8—C9—C10—O1	−177.6 (4)
N1—Fe1—N3—C20	−95.9 (3)	C8—C9—C10—C5	2.5 (7)
N2—Fe1—N3—C18	170.3 (3)	N2—C11—C12—C13	−177.5 (4)
N2—Fe1—N3—C20	−19.0 (3)	N2—C11—C12—C17	1.8 (6)
O1—Fe1—N5—C21	−103.2 (3)	C11—C12—C13—C14	179.6 (4)
O1—Fe1—N5—C23	77.9 (3)	C17—C12—C13—C14	0.2 (6)
O2—Fe1—N5—C21	1.8 (3)	C11—C12—C17—O2	1.8 (6)
O2—Fe1—N5—C23	−177.1 (3)	C11—C12—C17—C16	−179.7 (4)
N1—Fe1—N5—C21	167.6 (3)	C13—C12—C17—O2	−178.9 (4)
N1—Fe1—N5—C23	−11.3 (3)	C13—C12—C17—C16	−0.4 (6)
N2—Fe1—N5—C21	90.6 (3)	C12—C13—C14—C15	0.4 (7)
N2—Fe1—N5—C23	−88.3 (3)	C13—C14—C15—C16	−0.7 (7)
Fe1—O1—C10—C5	7.0 (6)	C14—C15—C16—C17	0.5 (7)
Fe1—O1—C10—C9	−172.9 (3)	C15—C16—C17—O2	178.6 (4)
Fe1—O2—C17—C12	−3.2 (6)	C15—C16—C17—C12	0.1 (6)
Fe1—O2—C17—C16	178.4 (3)	N4—C19—C20—N3	0.5 (5)
Fe1—N1—C2—C1	−165.7 (3)	N6—C22—C23—N5	−0.1 (5)

### Hydrogen-bond geometry (Å, °)

**Table:**

*D*—H···*A*	*D*—H	H···*A*	*D*···*A*	*D*—H···*A*
C7—H7···O6^i^	0.95	2.53	3.436 (6)	161
C16—H16···O5	0.95	2.53	3.325 (6)	142
N4—H4A···O2^ii^	0.88	2.48	3.063 (4)	125
N4—H4A···O6^ii^	0.88	2.36	3.031 (4)	133
N6—H6A···O4^iii^	0.88	2.03	2.892 (4)	167

Symmetry codes: (i) −*x*+1, −*y*, −*z*+2;  (ii) −*x*+2, −*y*, −*z*+2; (iii) −*x*+1, −*y*, −*z*+1.
